# Zelen design clinical trials: why, when, and how

**DOI:** 10.1186/s13063-021-05517-w

**Published:** 2021-08-17

**Authors:** Gregory E. Simon, Susan M. Shortreed, Lynn L. DeBar

**Affiliations:** Kaiser Permanente Washington Health Research Institute, Seattle, USA

**Keywords:** Zelen, Informed consent, Randomized trial

## Abstract

**Background:**

In 1979, Marvin Zelen proposed a new design for randomized clinical trials intended to facilitate clinicians’ and patients’ participation. The defining innovation of Zelen’s proposal was random assignment of treatment prior to patient or participant consent. Following randomization, a participant would receive information and asked to consent to the assigned treatment.

**Methods:**

This narrative review examined recent examples of Zelen design trials evaluating clinical and public health interventions.

**Results:**

Zelen designs have often been applied to questions regarding real-world treatment or intervention effects under conditions of incomplete adherence. Examples include evaluating outreach or engagement interventions (especially for stigmatized conditions), evaluating treatments for which benefit may vary according to participant motivation, and situations when assignment to a control or usual care condition might prompt a disappointment effect. Specific practical considerations determine whether a Zelen design is scientifically appropriate or practicable. Zelen design trials usually depend on identifying participants automatically from existing records rather than by advertising, referral, or active recruitment. Assessments of baseline or prognostic characteristics usually depend on available records data rather than research-specific assessments. Because investigators must consider how exposure to treatments or interventions might bias ascertainment of outcomes, assessment of outcomes from routinely created records is often necessary. A Zelen design requires a waiver of the usual requirement for informed consent prior to random assignment of treatment. The Revised Common Rule includes specific criteria for such a waiver, and those criteria are most often met for evaluation of a low-risk and potentially beneficial intervention added to usual care. Investigators and Institutional Review Boards must also consider whether the scientific or public health benefit of a Zelen design trial outweighs the autonomy interests of potential participants. Analysis of Zelen trials compares outcomes according to original assignment, regardless of any refusal to accept or participate in the assigned treatment.

**Conclusions:**

A Zelen design trial assesses the real-world consequences of a specific strategy to prompt or promote uptake of a specific treatment. While such trials are poorly suited to address explanatory or efficacy questions, they are often preferred for addressing pragmatic or policy questions.

## Introduction

Traditional randomized clinical trials often fail to provide the relevant and timely evidence necessary to guide clinical and policy decisions regarding medical treatments or services [1–4]. Regarding relevance, participants in traditional randomized trials often differ markedly from those treated in community practice in terms of sociodemographic characteristics, prognostic characteristics, co-occurring conditions, and motivation or likelihood of treatment adherence. Consequently, outcomes observed in clinical trial participants may differ from those in real-world practice where trial results would be applied [1, 5, 6]. Furthermore, narrow entry criteria, complex consent procedures, and burdensome research assessments can slow or reduce recruitment. Inadequate recruitment contributes to the slow pace and frequent failure of many traditional clinical trials [3, 4, 7, 8].

Concerns regarding the efficiency of evidence generation and the generalizability of resulting evidence have prompted demands for evidence derived from real-world settings using more pragmatic research designs [1, 3, 5, 6]. Pragmatic or real-world clinical trials may differ from traditional trials in several dimensions [5, 6], including the specific mechanism of allocating treatment conditions or study groups. Alternatives to traditional methods of patient-level recruitment and random assignment include cluster-level random assignment, stepped-wedge designs, and Zelen designs [9, 10]. Selection of the optimal method for treatment assignment depends on the specific question to be addressed and the practical or ethical constraints imposed by the trial setting and the treatment(s) under study. Motivated by increasing interest in real-world evidence and pragmatic clinical trials, this narrative review examines the Zelen design as one type of pragmatic trial, focusing on the specific questions and settings to which the Zelen design is best suited.

Here we review key aspects of the Zelen design, characterized by randomization prior to consent followed by encouragement to accept the assigned treatment. Variations of the Zelen design may be applied to a comparison of a new treatment to a no-treatment control condition or to a comparison of alternative “active” treatments. We describe the original motivation for this design and then consider four questions: For which questions might a Zelen design be preferred? Under what conditions is this design scientifically appropriate? When is this design ethically appropriate? How should results of a Zelen design trial be interpreted?

## Defining characteristics of the Zelen design

In 1979, Marvin Zelen proposed a new design for randomized clinical trials intended to facilitate clinicians’ and patients’ participation [9]. He argued that the traditional randomized trial design, requiring informed consent prior to treatment assignment reduced clinicians’ likelihood of recommending trial participation and patients’ likelihood of participating. Clinicians might be reluctant to acknowledge uncertainty about alternative treatments, and patients might prefer a new treatment presumed to be superior. In addition, comparisons of new treatments to standard care could be biased by disappointment effects when all participants are informed regarding a new treatment and only some receive it [11–13]. The defining innovation of Zelen’s proposal was random assignment of treatment prior to patient or participant consent. A comparison of alternative “active” treatments would follow a “double consent” design [13]. Following random assignment to alternative treatments, participants in both arms would receive information about and would be asked to consent to their assigned treatment. A comparison of some new treatments to a usual care or no-treatment control condition would follow a “single consent” design [13]. Participants assigned to the new treatment would receive information and asked to consent, while those assigned to no treatment or usual care would generally not be contacted or notified. In either design, analyses would compare outcomes according to the original treatment assignment, regardless of any refusal to accept or participate in the assigned treatment. Those who decline or discontinue the assigned treatment would be analyzed according to the initial assignment, preserving the unbiased assignment of the original randomization. Delaying informed consent until after randomization was expected to increase acceptability to clinicians and patients as well as reduce biases due to notifying a participant about a treatment they would not receive.

Zelen’s original proposal [9] acknowledged some important limitations of this design. Informed consent describing only the assigned treatment would not allow blinding of clinicians or patients. Patients’ expectations or preferences could bias subsequent assessment of outcomes. In addition, any true difference in efficacy or safety between treatments would be diluted by patients declining to receive the assigned treatment, a potential loss of precision. Zelen argued that any loss of efficiency could be overcome by increased rates of trial participation and subsequent increases in sample sizes.

Over the last 10 years, Zelen designs have been employed to evaluate a range of outreach and care navigation interventions to promote treatment engagement, including postcard or electronic messaging outreach to prevent suicide attempts or self-harm [14, 15], eHealth intervention to increase engagement in eating disorder treatment [16], care navigation to promote advanced care planning in older adults [17], and assessment plus feedback to improve early adherence to substance use treatment [18]. Similarly, this design has been used to evaluate health promotion or behavior change interventions, including a text message for HIV prevention [19], rehabilitation interventions for musculoskeletal conditions [20–22], health coaching to improve chronic illness management [23], and oral health intervention to prevent early childhood caries [24]. Zelen designs have also been employed to evaluate programs to prevent re-hospitalization [25] or reduce frequent emergency department use [26].

## When is the Zelen design preferred?

Zelen’s original proposal discussed this design as an alternative method for either assessing efficacy of new treatments or comparing the efficacy of existing treatments. Incomplete uptake of an assigned treatment was considered a limitation or a necessary accommodation to serve the goals of increased participation in clinical research. Zelen wrote, “If only a small proportion of patients are willing to take treatment B, this experimental plan may be useless in evaluation of this treatment.” [9]

For some research questions, however, allowing incomplete uptake of an assigned treatment may be a strength rather than a limitation. In pragmatic trials, the acceptability of a treatment or intervention is often a central determinant of overall effectiveness. The Zelen design can accommodate delayed or partial uptake of treatment, more accurately reflecting real-world variability. Assessing effectiveness under conditions of incomplete uptake or adherence is especially relevant to the evaluation of outreach or prevention programs or when evaluating interventions for stigmatized conditions. When stigma reduces uptake of or adherence to a treatment under study, the resulting decrease in population-level benefit is an important component of real-world effectiveness. In each of the Zelen design examples cited above, initial refusal of an assigned intervention or early discontinuation of that intervention would be considered signal rather than noise. In evaluating outreach, engagement, and health promotion interventions, research questions typically concern uptake in settings where the treatment would be implemented rather than potential efficacy if all participants behaved as investigators might hope.

Similarly, the Zelen design may be especially appropriate for evaluating intervention effects when both uptake and potential benefit are expected to vary according to participants’ motivation. For example, an outreach and care management intervention to reduce self-harm among high-risk outpatients [15] could have broad effects via nonspecific support to all who receive outreach messages as well as more specific effects among those who engage in recommended treatment. At the same time, outreach to promote engagement could actually reduce the use of recommended treatment in those who are upset or offended by outreach messages. In these situations, the effects of participants’ values and preferences on treatment uptake or adherence constitute signal rather than noise. A traditional randomized trial, limited to those who agree to participate in a trial of outreach and engagement, could not accurately assess the net effect of these different processes.

In addition, a Zelen design may be preferred when assignment to a no-treatment or usual care control condition could prompt a disappointment effect. In that case, outcomes of those assigned a control condition may not accurately reflect what would have occurred in the absence of trial participation. Even when explicitly informed that a new treatment may have no benefit, many clinical trial participants presume that a new treatment will be superior to current practice [27]. Therapeutic optimism regarding a new or “experimental” treatment is mirrored by disappointment effects in those assigned to usual or standard care. In a single consent design evaluating a new treatment, disappointment at being assigned to a “control” condition could reduce participation in outcome data collection or influence participants’ reporting of study outcomes. In a double consent design comparing alternative treatments, disappointment over assignment to a less preferred treatment could artificially reduce adherence. Minimizing this disappointment bias was an important motivation for Zelen’s original proposal [9] that patients assigned to a usual care control condition would not be notified regarding the potential benefits of a treatment they could not actually receive. A fundamental principle of intent-to-treat analyses in a Zelen design is that incomplete uptake or adherence are key components of effectiveness, so those who decline an assigned treatment should be analyzed according to the original assignment. But a disappointment effect due to notification regarding alternative treatment assignments offered to others is an artifact of the research process. Consequently, the consent process in a Zelen design only considers the assigned treatment and does not include information regarding treatments not being offered.

## When is the Zelen design scientifically appropriate?

Even when a Zelen design might be preferred to address a specific clinical or policy question, practical considerations will influence whether such a design is scientifically appropriate or practicable. Relevant aspects of the trial design include identification of potential participants, assessment of relevant baseline or prognostic characteristics, evaluating quality or fidelity of treatments, and potential bias in the ascertainment of outcomes.

In general, the use of a Zelen design requires that participants can be identified automatically rather than recruited via advertising or referral. This might be accomplished using health system records, educational records, social service records, or some other administrative data source. In any case, eligibility or inclusion must be definitively determined prior to randomization. For example, recent Zelen design trials have automatically enrolled patients from a defined population using records of hospital discharge [25] or routinely administered mental health questionnaires [15]. Recruitment procedures typical of traditional randomized trials are not consistent with the goal of including all eligible to receive an intervention, regardless of motivation or expected participation. Identification of participants via community advertising or clinician referral would usually select participants more likely to accept and adhere to the treatments under study [28].

Similarly, assessment of baseline or prognostic characteristics must be limited to information available from existing records for all randomized participants. Limiting randomization to those willing to complete a research-specific assessment would be inconsistent with the goal of assessing intervention or treatment effects in all who are eligible. Assessing baseline or prognostic characteristics after randomization raises the risk of significant bias if either participation in a research assessment or responses to a research assessment might be influenced by a patient’s knowledge of treatment assignment.

For similar reasons, comparison of treatment adherence or service utilization after random assignment must sometimes be limited to information available from existing records. This concern is especially relevant in trials of outreach programs to promote treatment engagement or improve treatment adherence. For participants assigned to the offer of a new treatment or program, it is essential to examine uptake or adherence among all those assigned, rather than limiting assessment to those who accept the offered treatment or those willing to participate in research assessments. If treatment uptake or adherence are assessed using research assessments, then participation in those assessments could certainly be affected by exposure to intervention(s) under study, introducing a significant potential bias. In addition, contacting participants or clinicians assigned to usual care (i.e., no additional outreach) control group could influence care delivery so that it no longer resembled care as usual.

Finally, investigators must consider how exposure to treatments or interventions under study might bias ascertainment of outcomes. In a comparison of some new service or program to a no-treatment or usual care control group, the offer of that new intervention could influence likelihood of participation in research outcome assessment. The resulting differential availability of outcome information could introduce significant bias. Consequently, a Zelen design is most appropriate when the primary outcome is a well-defined event that can be ascertained on all individuals randomized from automatically collected data, such as health system records, vital statistics data, educational records, or social service records. Examples of outcomes from health records include emergency department visit [26], hospitalization [25], routinely administered patient-reported outcome questionnaires, or a hospital clinician’s diagnosis of probable suicide attempt [14, 15].

## When is the Zelen design ethically appropriate?

In a traditional clinical trial, a single-informed consent process encompasses random assignment, receipt of study treatment(s), and participation in study assessments. Potential participants receive information regarding all possible treatments, including both “active” and control treatments, and are asked to consent to any treatment that is subsequently assigned. In other words, informed consent for participation in traditional clinical trials is typically indivisible; potential participants agree to all aspects of a trial prior to randomization, or they do not participate at all.

Randomization prior to informed consent is a central feature of the Zelen or randomized encouragement design. Each participant receives information regarding only the treatment to which they have been assigned. Zelen’s original description allowed variation in this consent process, depending on treatments under study. In a comparison of some treatment or service with usual care, those assigned to be offered the new treatment or service would receive information and be allowed the opportunity to accept or decline. Those declining would receive care as usual. In this scenario, those assigned to receive care as usual would receive information as in usual practice, with no formal research consent procedure. In a comparison of alternative active treatments, each participant would receive information regarding the treatment to which they were assigned and allowed the opportunity to accept or decline. Those declining would then receive care as usual. Either of these variations would require a waiver of the usual requirement for informed consent to be randomly assigned.

Under the revised Common Rule [29] governing research supported by US government agencies, the requirement for informed consent prior to random assignment can be waived if specific criteria are met. First random assignment to usual care or to an “active” treatment under study must not create more than minimal additional risk. Second, waiver of informed consent prior to randomization must be necessary for the research to be practicable. Third, waiver of consent for random assignment must not adversely affect the rights or welfare of participants. Fourth, when appropriate, participants should be provided with additional pertinent information after randomization.

These requirements for waiver of consent for random assignment are most often met when a Zelen design is used to evaluate the benefit of some potentially beneficial treatment or service added to usual care. Regarding the “practicability” criterion, Zelen argued that randomization prior to consent is sometimes essential for valid evaluation of treatment effectiveness, especially when incomplete uptake is likely or “disappointment effects” might distort outcomes in participants assigned to usual care. Regarding other criteria for waiver of consent, investigators and Institutional Review Boards (IRBs) should separately evaluate impacts on participants assigned to usual care or to some “active” intervention. Each participant assigned to usual care receives precisely the same treatment they would have received if not included in the trial. By definition, assignment to the care one would ordinarily receive does not involve any increase in risk nor adversely affect a potential participant’s rights or welfare – as long as that assignment does not involve any restriction on the use of services normally available. Following Zelen’s original argument, notifying a participant of their assignment to usual care rather than some new treatment or service would not usually be appropriate or useful. That notification would offer no additional benefit or protection and could cause harm by provoking a disappointment effect. For a participant assigned to be offered some new treatment or service, we should distinguish between the effects of offering that treatment and the effects of using or receiving it. As discussed above, any participant offered some study treatment or service would subsequently be provided information and given the opportunity to accept or decline. When applying revised Common Rule criteria, we should therefore consider how assignment to an offer of a study treatment might create more than minimal risk or adversely affect a participant’s rights or welfare. Satisfying the “minimal risk” criterion usually requires that the potential risks of a study treatment be reasonably well established. Satisfying the “rights and welfare” criterion requires that the offer of a study treatment be transparent (i.e., acknowledges that the treatment or service being offered is part of research), non-coercive (i.e., clearly communicates right to refuse or withdraw), and not involve any restriction on access to treatment normally available. Allowing free choice to accept or decline an offered treatment is consistent with both the scientific aims of a Zelen design and the ethical obligations of researchers.

Applying regulatory requirements for waiver of consent is more complex for trials involving random assignment to alternative active treatments. Zelen’s argument still applies regarding the “practicability criterion”; random assignment prior to the offer of treatment is sometimes necessary to accurately assess real-world effectiveness and avoid disappointment effects. In such a design, all participants would be offered additional information after randomization, when the assigned treatment is offered. Applicability of the “minimal risk” and “rights and welfare” criteria depends on the specifics of the treatments under study and the options available to those who decline the offer of an assigned treatment. To satisfy criteria for waiver of consent, the offer of assigned treatment must be transparent and non-coercive. In addition, assigned treatments or programs should not involve any restrictions on the use of treatments normally available. In a trial comparing treatments in common use, satisfying these criteria may require that a participant be permitted to “cross over” and receive the alternative treatment under study. A pre-consent random assignment that restricted or denied access to a treatment normally available would likely not satisfy the “rights and welfare” criterion of the Revised Common Rule. Restricting access to a treatment usually available would be most concerning when random assignment determines choices about which patients may have strong preferences. In that case, informed consent prior to random assignment would usually be expected.

Simply satisfying regulatory requirements for waiver of informed consent does not necessarily imply that such a waiver is ethically appropriate. Investigators and IRBs considering such a design should consider the basic ethical principles described in the Belmont Report [30], specifically the principles of beneficence and respect for persons. Regarding beneficence, investigators are expected to maximize potential benefits to participants and minimize potential harms. Those considerations should influence the selection of study eligibility criteria, design of study interventions or treatments, design of intervention or consent procedures, and design of procedures for any study-specific assessments or data collection. Regarding respect for persons, random assignment of potential participants prior to any notification or consent does infringe on the principle that “individuals should be treated as autonomous agents.” Even if random assignment to be offered or not offered a new treatment creates no risk or harm, many patients might expect to be involved in that decision [31]. Consequently, investigators and IRBs must consider whether the scientific and public health benefits of randomization prior to consent outweigh infringing on the autonomy of potential participants.

Zelen’s proposal presumed a traditional informed consent process at the time any study treatment is offered. That process would typically include all essential elements of informed consent: explicit notification that the offer is a research activity, a description of expected procedures, a description of potential benefits and risks, notification that participation or acceptance is voluntary, and a description of alternatives to the treatment offered. For some treatments or services, however, an abridged consent procedure(15) or a waiver of informed consent to receive a study treatment might be appropriate. Such a waiver would be permissible if all of the regulatory criteria above are satisfied regarding receipt of the study treatment. Satisfying the “practicability” criterion would require a convincing argument that the research would not be practicable if a traditional informed consent procedure were required for those assigned to “experimental” treatment.

Use of a Zelen design usually requires research use of healthcare or other administrative records without explicit consent or authorization. Such data are often necessary to select eligible patients or potential participants prior to any direct contact. In most cases, identified or identifiable data are necessary. That use of identifiable data is necessary and would be considered research involving human subjects. Investigators and IRBs would then need to apply the criteria listed above regarding waiver of consent for use of records data. In most cases, that use of records data would not create additional risk or adversely affect rights and welfare.

## How should findings of a Zelen design trial be interpreted?

Zelen’s proposal specified that analyses of Zelen trials should follow original treatment assignment or intent to treat [9, 10]. Any “as treated” or “per protocol” analysis would be irreparably biased. In a comparison of a new treatment or service with usual care, it is not possible to identify those in the usual care control group who would have accepted treatment had it been offered. In a comparison of alternative active treatments, it would still be problematic to compare those in each group accepting the offered treatment. We cannot assume that participants accepting one treatment would be otherwise similar to those who accept an alternative treatment.

Consequently, findings from the Zelen design studies focus on population effects and address questions regarding an offer of care. This design is optimal for assessing the overall benefits and harms of offering a treatment or service to the entire population of people eligible to receive it. Such questions are especially relevant for health system leaders considering the potential overall benefit of providing a particular healthcare program, particularly those for stigmatized conditions like substance use disorders, chronic pain, or mental health, in which eligible patients may be initially ambivalent at best about engaging in treatment.

Zelen did, however, propose that results observed under conditions of incomplete intervention uptake could be extrapolated to the full population meeting study eligibility criteria [9, 10]. This interpretation presumes that benefits or harms of the treatment under study would be similar in those who decline the treatment and those who accept it. In other words, extrapolating benefits or harms observed in a Zelen design trial to all those in the eligible population assumes that willingness to accept treatment does not modify differences between treatments in either benefits or harms.

We caution that overall benefits or harms observed in a Zelen design trial should not be adjusted for noncompliance or extrapolated to hypothetical populations with different rates of treatment uptake. We cannot presume that treatment effects observed in treatment “compliers” would be similar in those who decline an offered treatment or those who prematurely discontinue treatment. Consequently, average results observed in a Zelen design trial do not reflect a diluted or attenuated version of what would be observed if all participants accepted the offered treatment. In other words, “complier causal effects” [32, 33] would not necessarily generalize to those who receive the treatment of interest in the absence of intervention or those who decline the treatment despite intervention. Instead, findings only apply to patients at the margin [34], the middle group of patients who accept the study treatment if invited or encouraged but who would not receive it in usual care. For example, the Zelen design has been used to evaluate automatically offering smoking cessation services to all hospitalized smokers [35]. Any reduction in smoking rates observed in those who accept that offer cannot be extrapolated to those who decline. In other words, this design does not address the question “Does participation in this smoking cessation program increase quit rates?”. Instead, it addresses the question “Does a policy of routinely offering this smoking cessation program increase overall quit rates?”

Furthermore, findings of any specific Zelen design trial may not generalize to some other form of invitation or encouragement. Even if alternative forms of invitation or encouragement lead to similar rates of treatment uptake, those accepting different forms of invitation might differ with respect to benefits or harms of any specific treatment. In addition, an invitation or offer of treatment may have nonspecific supportive effects. The Zelen design does not support distinguishing specific effects of a study treatment from non-specific effects of support or encouragement.

Just as designs focused on efficacy may not accurately assess real-world effectiveness, a Zelen design trial well-suited to evaluate effectiveness may not accurately assess efficacy. Instead, a Zelen design assesses the real-world consequences of a specific strategy to prompt or promote uptake of a specific treatment. While such trials are poorly suited to address explanatory or efficacy questions, they are often preferred for addressing pragmatic or policy questions.

## Data Availability

N/A
